# Sequential Delivery of Dual Growth Factors from Injectable Chitosan-Based Composite Hydrogels

**DOI:** 10.3390/md17060365

**Published:** 2019-06-20

**Authors:** Qing Min, Jiaoyan Liu, Xiaofeng Yu, Yuchen Zhang, Jiliang Wu, Ying Wan

**Affiliations:** 1 School of Pharmacy, Hubei University of Science and Technology, Xianning 437100, China; baimin0628@hbust.edu.cn (Q.M.); ningwu1985@163.com (Y.Z.); 2 College of Life Science and Technology, Huazhong Universityf of Science and Technology, Wuhan 430074, China; liujiaoyan@hust.edu.cn (J.L.); m201771729@hust.edu.cn (X.Y.)

**Keywords:** microspheres, growth factor, composite hydrogel, sequential delivery, platelet-derived growth factor-BB, bone morphogenetic protein-2

## Abstract

Local administration of platelet-derived growth factor-BB (PGDF-BB) and bone morphogenetic protein-2 (BMP-2) in a sequential release manner could substantially promote bone healing. To achieve this goal, a delivery system that could sustain the release of PGDF-BB and BMP-2 by way of temporal separation was developed. One type of PGDF-BB-encapsulated alginate microsphere and another type of BMP-2-encapsulated microsphere with a core-shell structure were respectively produced using emulsification methods. These two types of microspheres were then embedded into chitosan/glycerophosphate hydrogel for constructing composite gels. Some of them were found to be injectable at ambient temperature and had thermo-sensitive features near physiological temperature and pH. The optimally formulated composite gels showed the ability to control the release of PGDF-BB and BMP-2 in a sequential fashion in which PDGF-BB was released earlier than BMP-2. In vitro release patterns indicated that the release rates could be significantly regulated by varying the embedded amount of the factor-encapsulated microspheres, which can in turn mediate the temporal separation release interval between PGDF-BB and BMP-2. The released PDGF-BB and BMP-2 were detected to be bioactive based on their respective effects on Balb/c 3T3 and C2C12 cells. These results suggest that the presently developed composite gels have the potential for bone repair by synergistically utilizing the early chemotactic effect of PDGF-BB and the subsequent osteogenic and angiogenic functions of PDGF-BB and BMP-2.

## 1. Introduction

Bone tissue has certain self-healing abilities after an injury, provided that the impairment is mild and the defect size is small [1,2]. However, in cases where large-size bone defects occur, bone grafts are usually needed for bone repair [1,2]. Clinical approaches to repairing large bone defects often involve the utilization of autografts and allografts, but these treatments have their own disadvantages [3,4]. Nowadays, tissue engineering technique has emerged as an option for bone repair [5,6,7,8,9,10]. 

Local administration of growth factors to promote bone repair has achieved promising results in many preclinical and clinical models [6,7,10,11,12]. Platelet-derived growth factor (PDGF) and bone morphogenetic protein-2 (BMP-2) are commonly used growth factors for bone repair [10,11]. BMP-2 has been utilized for the treatment of tibial fractures and for the lumbar spinal fusion by employing absorbable collagen sponge (ACS) as a carrier [6,7,12]. Although the BMP-2-incorperated ACS set is clinically viable for fracture repair, its drawbacks can result in hypertrophic or atrophic non-unions [13,14]. In addition, BMP-2 is currently administered in dosages much greater than physiological concentrations, which makes such treatments costly and increases the likelihood for ectopic bone formation [6,11,13,14].

The utility of a single factor may enhance bone healing by inducing osteogenesis and angiogenesis [15]. Increasing evidence reveals that bone healing could be substantially improved via the appropriate administration of growth factors in certain combinations [6,7,16]. The simultaneous use of two kinds of growth factors is one of several commonly implemented strategies for enhancing bone repair [6,7,16,17]. However, there is other evidence that this strategy may not result in desired outcomes in many cases [16,17]. It has been recognized that bone healing is an evolutionarily conserved, complex multicellular, and multi-stage process [17]. Varied kinds of secreted growth factors are found to be present in the bone healing microenvironment in a cascading fashion to coordinate the stages of bone regeneration [18]. When specifically considering the temporal roles of different growth factors, it should be more viable to administer the applied growth factors in a sequential rather than simultaneous manner. 

PDGF-BB is known for its role in the recruitment of mesenchymal stem cells and other precursor cells to the applied site, and it also has the capacity to stimulate VEGF production, which in turn promotes blood vessel formation and regulates angiogenesis of neonatal bone tissue [19,20,21]. Accordingly, it could be beneficial for bone repair by using PDGF-BB and BMP-2 together by way of temporal separation. Based on the functions of PDGF-BB and BMP-2, PDGF-BB release should start at the earlier stage of bone repair, allowing it to recruit cells from the surrounding area of the applied site and to initiate the occurrence of blood vessel formation; and on the other hand, BMP-2 delivery can be delayed to facilitate the establishment of a mature vascular network and to promote the organized bone regeneration. 

Injectable hydrogels have advantages for the delivery of drugs or active agents because they can localize them to the defect sites via minimal invasive injection while conveniently filling complex defects [22]. To date, hydrogels based on natural polymers have attracted a lot of attention in the very volume of medical applications. Alginate (ALG) is a natural polysaccharide and has been extensively investigated for different biomedical applications in the form of hydrogels and microspheres (MPs) that are usually prepared using some divalent ionic crosslinkers, such as Ca^2+^, Sr^2+^, and Zn^2+^ [23,24]. Chitosan (CH) is another kind of natural polysaccharide and has been widely used for various biomedical purposes owing to its many advantages [25,26,27,28,29]. Among chitosan-based hydrogels, chitosan (CH)/glycerophosphate (GP) hydrogel has received strong interest because of its injectable and thermo-sensitive features [25]. Although CH/GP gel has the potency to serve as an carrier for delivering growth factors, the direct incorporation of growth factors into CH/GP gel would result in burst factor release due to the porous structure and high water content of the gel [21,22]. 

In this study, an attempt was made to develop hydrogel systems for sequentially delivering PDGF-BB and BMP-2. PDGF-BB was loaded into a type of alginate microsphere, and separately, BMP-2 was loaded into another type of core-shell microsphere. Two types of microspheres were embedded into CH/GP hydrogel for constructing composite gel systems through which PDGF-BB can be released earlier and BMP-2 would be released in a temporally separated manner. Some composite gels were found to show a well-controlled capacity to administer the sequential release of PDGF-BB and BMP-2 while effectively preserving their bioactivity. 

## 2. Results and Discussion 

### 2.1. Parameters of Microspheres

**Figure 1:**
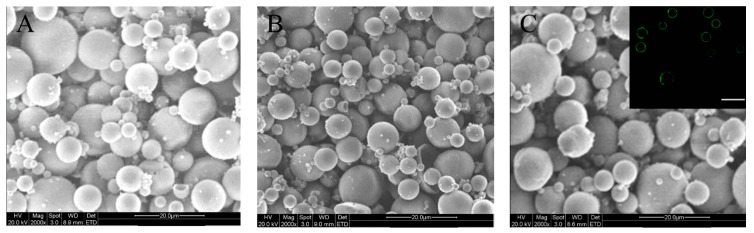
SEM images of blank alginate (ALG) microspheres (MPs) (**A**); blank chitosan-polylactide (CH-PLA) MPs (**B**); and blank core-shell MPs (**C**; inserted fluorescent image denotes the shell layer of MPs; scale bar: 30 μm).

Ca^2+^-crosslinked ALG MPs are often used for delivering protein factors since they are able to retain their bioactivity well [23,24,26]. In addition, the release pattern of the loaded factor can be regulated by majorly changing the applied amount of Ca^2+^ ions [23,26]. In this study, MPs with potency in injectable applications are needed for administering the release of PDGF-BB and BMP-2 in a sequential fashion in which PDGF-BB was released earlier than BMP-2. Hence, ALG MPs were employed as a carrier for delivering PDGF-BB. Blank ALG MPs were first produced to optimize their size and crosslinking in order to save the costly PDGF-BB. An emulsification method was used to prepare blank ALG MPs by controlling two major parameters: the concentration of ALG solutions and the amount of used CaCl_2_. The optimal compositional proportion and processing conditions for these MPs are provided in the experimental section. Figure 1A shows a representative SEM image for the blank ALG MPs. The image exhibits that these MPs had good sphericity and smooth surfaces with varied sizes ranging from several microns to around 20 microns.

By grafting hydrophobic polylactide (PLA) oligomers onto the CH backbone, the resulting chitosan-polylactide (CH-PLA) copolymers could be soluble in water-based solvents while having some merits stemmed from CH and PLA [30,31]. To this end, a series of CH-PLAs with varied PLA weight percentages was synthesized by selectively grafting PLA to the C-6 sites of CH units and leaving the amino groups of the CH backbone free for subsequent crosslinking. CH-PLA with a PLA content of around 31 wt% was selected for the preparation of CH-PLA MPs in view of the solubility of CH-PLA in 1.0% acetic acid aqueous solutions. Blank CH-PLA MPs were also prepared with an emulsification method by using sodium tripolyphosphate (TPP) as a crosslinker considering the good in vivo safety of TPP [32]. Figure 1B presents a SEM image for the blank CH-PLA MPs. These MPs are seen to be spherical and have their size less than 20 μm. Several sets of blank ALG MPs and blank CH-PLA MPs were measured for their mean size and zeta (ζ) potential, and the obtained data are listed in Table 1. It can be observed from Table 1 that in contrast to BM-I MPs, BM-II MPs had a mean size of around 8 μm, which is significantly less than that of BM-I. Additionally, BM-II MPs exhibited a positively charged surface feature, which is indicated by their high positive ζ potential. As mentioned earlier, the factor-encapsulated MPs need to be embedded into the CH/GP gel for constructing composite gels. A concern was thus found to arise from the positively charged surface property of BM-II MPs because our tentative experiments revealed that the BM-II-embedded composite gels had a pronouncedly elevated gelling temperature and considerably prolonged gelling time, making these gels inapt for the potential use in the clinic. 

ALG MPs had a negative ζ potential, and accordingly, it would be feasible to alter the surface charging nature of CH-PLA MPs by coating the CH-PLA MPs with an ALG layer in the light of anionic features of ALG. In order to endow the blank ALG-coated CH-PLA MPs with a mean size similar to that of the ALG MPs, an attempt was made to prepare smaller blank CH-PLA MPs so that they could become similar to ALG MPs in mean size after being coated. Data in Table 1 indicate that the blank ALG-coated CH-PLA MPs indeed had a mean size very similar to that of ALG MPs without significant difference (*p* > 0.05), and meanwhile, their ζ potential also became similar to that of ALG MPs (*p* > 0.05). Figure 1C shows a SEM image for BM-III MPs. These MPs bear similarity to BM-I MPs in size and morphology, and clearly, the inserted fluorescent image further demonstrates the presence of a coating layer on the surface of these MPs. 

Table 1 shows that BM-I MPs had a large swelling index (SI) whereas BM-II MPs and BM-III MPs had much smaller SI. As described in the experimental section, BM-II MPs were prepared using TPP as an ionic crosslinker, and the CH component in the BM-II MPs has hydrophilic and swelling nature. As a result, BM-II MPs could be swollen to some extent because the TPP-connected network in the MPs could have become loose when the MPs were exposed to an aqueous environment. On the other hand, the CH-PLA used for preparing BM-II MPs contains around 31 wt% of PLA. Hence, a large number of PLA side chains in CH-PLA molecules would entangle CH-PLA chains together to prevent the MPs from swelling because PLA is highly hydrophobic and non-swelling in water [33]. Therefore, the relatively small SI for BM-II MPs can be mainly ascribed to the effect of the PLA component in these MPs. When compared with BM-II MPs, BM-III MPs have an ALG coating layer, but this layer could exert a very limited impact on SI of BM-III MPs because the coating layer is very thin (see Figure 1C) and has been hardened by Ca^2+^ ions. Accordingly, BM-III MPs have their SI similar to that for BM-II MPs without significant difference (*p* > 0.05). In the case of BM-I MPs, although they were crosslinked by Ca^2+^ ions, the employed ALG component is water-soluble, and their ALG chain network built by ionic linkages would be loose in a wet sate; these two factors would jointly lead to a large SI for BM-I MPs.

**Figure 2:**
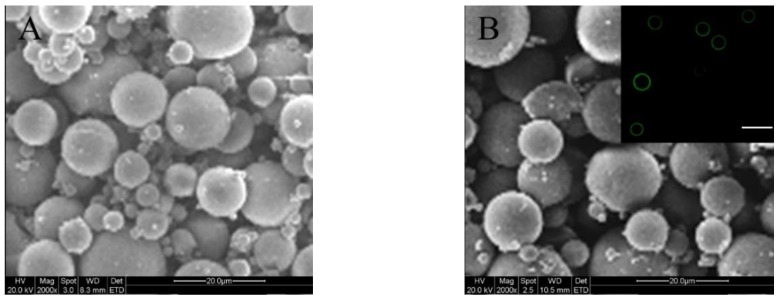
SEM images of platelet-derived growth factor-BB (PDGF-BB)-encapsulated ALG MPs (**A**); and bone morphogenetic protein-2 (BMP-2)-encapsulated core-shell MPs (**B**; inserted fluorescent image denotes the shell layer of MPs; scale bar: 30 μm).

On the basis of investigations for blank MPs, PDGF-BB and BMP-2 were separately loaded into ALG MPs and ALG-coated CH-PLA MPs, using the same methods and formulations respectively applied to the preparation of BM-I and BM-III MPs, in order to achieve the desired MPs with a mean size and electrical surface property similar to their corresponding blank MP counterparts. Some results for these factor-loaded MPs are presented in Figure 2 and Table 2. The two SEM images signify that both kinds of factor-loaded MPs had their size and morphology similar to their blank MP counterparts. Figure 2B reveals that the coating layer of these MPs is thin and looks similar to that for BM-III MPs, their blank counterpart (see Figure 1C). Data in Table 2 indicate that the addition of growth factor into these MPs seems to not exert significant impacts (*p* > 0.05) on their mean size, ζ potential, and SI when compared to their corresponding blank MP counterparts. These results are reasonable, since the same preparation methods and the same formulations that were applied to their respective blank MP counterparts were employed, and in addition, the mass of the encapsulated PDGF-BB or BMP-2 in these MPs is very low in comparison to the mass of the MP matrices themselves.

It was found that the encapsulation efficiency (EE) for factor-loaded MPs was significantly affected by the fed amount of factor. The EE of factor-loaded MPs was therefore optimized by altering the fed amount of factor while keeping the composition of MPs and the preparation conditions constant to achieve possibly higher EE. The obtained EE for two types of MPs is shown in Table 2. It can be seen that LM-1 MPs had an EE of around 59%, which is much lower than that for LM-2 MPs. The difference in EE between LM-1 and LM-2 MPs is understandable if more details for these MPs are revealed. It is known that ALG is a water-soluble polysaccharide, and hence, a certain amount of PDGF-BB in the superficial layer of LM-1 MPs would be easily washed away during the preparation of LM-1 MPs even though these MPs were crosslinked by Ca^2+^ ions. Unlike LM-1 MPs, LM-2 MPs are composed of a core formed by BMP-2-encapsulated CH-PLA MPs and a shell formed by an ALG layer. Inside their core CH-PLA MPs, PLA branched chains in the CH-PLA component are hydrophobic and could be shaped as rigid hooks for griping BMP-2 molecules and CH-PLA chains together to form entanglements during the preparation of CH-PLA MPs. These entanglements would certainly prevent the loss of BMP-2 and result in a high EE for the CH-PLA MPs. The ALG coating layer for the BMP-2-encapsulated CH-PLA MPs was solidified by Ca^2+^ ions, and this layer would further act as a barrier to prevent BMP-2 molecules from being washed away during the preparation of LM-2 MPs. Consequently, LM-2 MPs have a high EE for loading BMP-2. 

### 2.2. Gelation Properties of Hydrogels without Factor Load 

In view of the similarity in sizes and ζ potential between the factor-loaded MPs and their blank MP counterparts, a series of MP-embedded composite gels was first prepared by embedding BM-I and BM-III MPs into the CH/GP gel in order to save the costly factors, and the resulting gels were used for evaluation of composite gels. CH/GP gel is known to have thermo-sensitive features near physiological temperature and pH [25], and the amount of MPs inside the composite gels was thus regulated within a proper range to retain the thermo-sensitive properties of the composite gels. 

**Figure 3:**
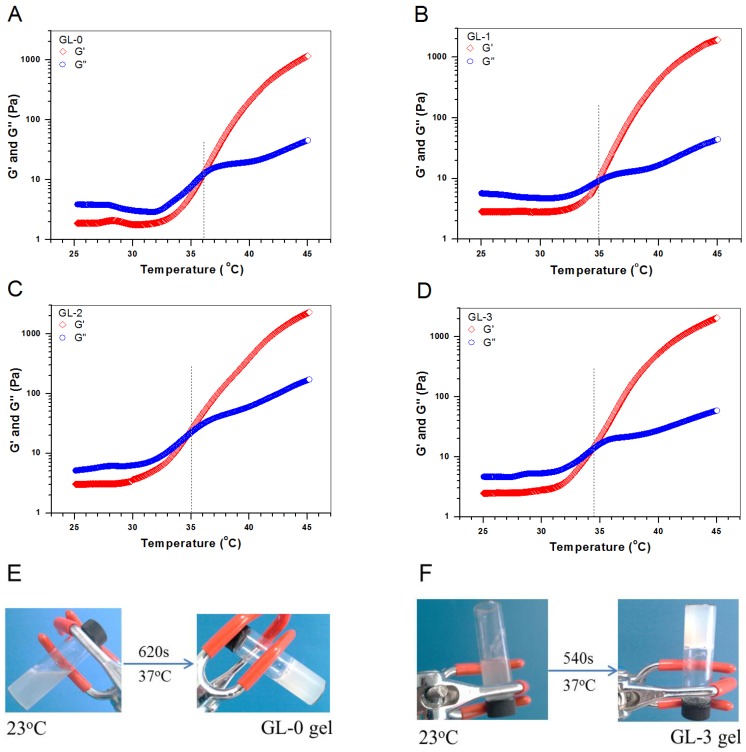
Temperature-dependence functions of G′ and G″ for four kinds of gels (**A**–**D**) without loading any growth factor. Representative sol-gel transition images for GL-0 (**E**) and GL-3 (**F**) gels (treatment temperature: 37 °C; and gelation time was estimated by tilting or inverting the vial every 20 s).

Figure 3 shows variations of modulus versus temperature for several gels as well as the typical sol-gel transitions for two kinds of gels. T_i_ for GL-0 gel was around 36 °C, whereas T_i_ for GL-1, GL-2, and GL-3 gels was around 35, 35.2, and 34.3 °C, respectively, suggesting that the gelling temperature of composite gels does not significantly change when compared to that for CH/GP gel. Figure 3F shows that the gelling time of GL-3 gels became significantly shorter than that of GL-0. Several sets of gels were measured to determine their pH, T_i_, and gelling time, and the obtained results are summarized in Table 3. Data listed in Table 3 indicate that the MP-embedded gels had a pH and T_i_ that is very similar to that for CH/GP gel without significant differences (*p* > 0.05). These results demonstrate that MP-embedded composite solutions are capable of forming into gels near physiological temperature and pH. Table 3 shows that GL-1, GL-2, and GL-3 had significantly shorter gelling times when compared with GL-0; the difference in gelling time could be attributed to the concentration effect of the employed composite solutions. It can be seen that the total amount of MPs embedded into GL-1, GL-2, and GL-3 gels was the same, and it is comparable to the amount of CH, meaning that the composite solutions used for preparing GL-1, GL-2, and GL-3 gels had the same concentration but were significantly thicker than that used for the preparation of GL-0 gel. Taking account of the commonness of gel matrix for all these gels, it can be inferred that the shortened gelling time for GL-1, GL-2, and GL-3 gels should be correlated to the increased concentration of the corresponding composite solutions when compared to GL-0 gel.

In principle, an injectable gel needs a fast sol-gel transition with durative stability because otherwise it could transude from the applied site and randomly migrate from one site to another, leading to unwanted effects [22]. On the other hand, it usually takes time to prepare the injectable mixture if the gel is used to load cells or drugs, which requires the resulting mixture to maintain good fluidity prior to gelling for easy injection [34]. In the present instance, the MP-embedded gels should be applicable for injection applications because their gelling time is only about 2 min shorter than that of the CH/GP gel, and the latter has been largely investigated for a variety of injection applications [22,25,29].

### 2.3. In Vitro Release 

**Figure 4:**
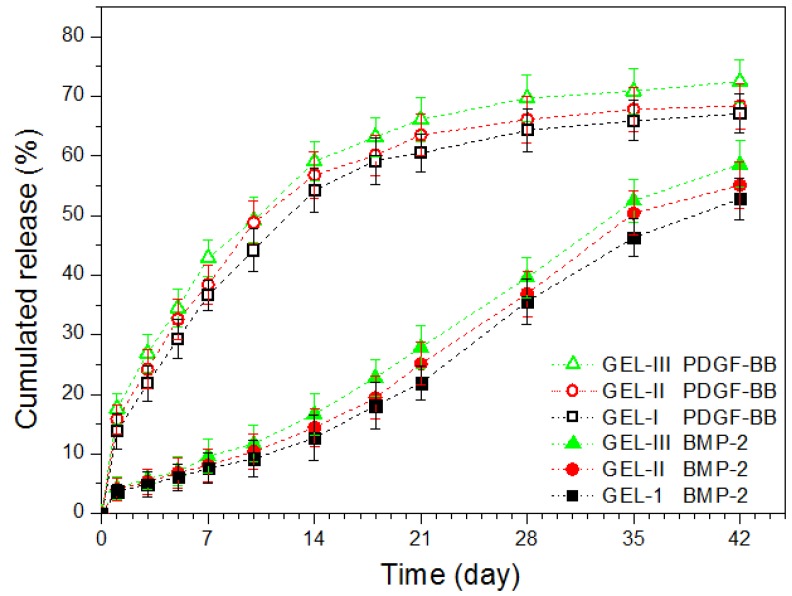
Cumulative release profiles for composite gels containing different amounts of growth factors but the same amount of microspheres.

Based on the results illustrated in Table 3, MP-embedded gels loaded with varied amounts of PDGF-BB and BMP-2 were prepared using the protocol similar to that applied to the preparation of GL-3 gel. Two kinds of MPs respectively loaded with PDGF-BB and BMP-2 were embedded into the CH/GP gel in two different ways, as formulated in Table 4 and Table 5. Release patterns for three kinds of gels that were loaded with different amounts of growth factors but the same amount of MPs are shown in Figure 4. The curves for PDGF-BB show that more than 10% of PDGF-BB was released in the first day, and at the end of two weeks, around 55% of PDGF-BB was released from these gels. In contrast to the patterns for PDGF-BB, BMP-2 was released from the gels in a considerably delayed way. Less than 10% of BMP-2 was released during the first week and the cumulative release amount of BMP-2 reached about 50% at the end of five weeks. These results reveal that such produced composite gels meet an important design requirement, namely the temporal presentation of the released PDGF-BB and BMP-2.

In principle, the release of drugs or bioactive reagents from polymeric matrices usually is correlated to swelling, diffusion, swelling followed by diffusion, and erosion [35]. In the present situation, the factor release was mediated by both MPs and the gel matrix. As shown in Table 4, these gels had the same gel matrix, and thus, the difference in the release behavior of PDGF-BB and BMP-2 should be critically ascribed to the effect of their corresponding MPs. PDGF-BB loaded ALG MPs had high SI, as shown in the example (see LM-1 MPs) in Table 2, due to the presence of loose ionic linkages among ALG chains when these MPs were exposed to the aqueous media. The Ca^2+^ ion crosslinked chain network inside the MPs thus has a limited ability to resist the loaded PDGF-BB molecules from being rapidly defused into the gel matrix, which would lead to high initial burst and subsequent fast release of PDGF-BB. In contrast to PDGF-BB, BMP-2 inside the core-shell MPs faces a rather different environment. As described in the experimental section, BMP-2 was first encapsulated into the CH-PLA MPs, and the resulting MPs were then coated by ALG. The CH-PLA chains in the core CH-PLA MPs have highly hydrophobic and degradation-tolerant PLA branches, and hence, they would grip BMP-2 molecules to form certain entanglements, preventing BMP-2 from getting out of the MPs. Moreover, the BMP-2 molecules that are detached from the core CH-PLA MPs have to be transported cross the ALG coating layer to reach the gel matrix. Accordingly, the double resistance stemmed from hydrophobic PLA branches inside the CH-PLA MPs and the barrier layer on the surface of the core-shell MPs would greatly hinder the diffusion of BMP-2 into the gel matrix, resulting in remarkably delayed release of BMP-2 from the composite gels.

The gels illustrated in Table 4 were produced by embedding two types of MPs at a fixed weight ratio while being endued with various loads of PDGF-BB and BMP-2 in attempt to regulate the release rate of the factors, since an increase in the factor load may facilitate the factor to diffuse out of the MPs and possibly result in faster factor release from these gels. However, it can be observed from Figure 4 that the initial factor load in these gels seemed to not have substantial impacts on their release profiles. A possible reason could be attributed to the factor-loaded MPs themselves. Data in Table 2 show that LM-1 and LM-2 MPs have capacities in carrying a high amount of PDGF-BB or BMP-2, respectively. The presently employed ALG MPs and core-shell MPs for the composite gels were loaded with much lower amounts of PDGF-BB and BMP-2 when compared to LM-1 and LM-2 MPs (see Table 2 and Table 4), respectively, since such loads for PDGF-BB and BMP-2 are enough for their applications due to the greatly reduced initial release for PDGF-BB and greatly delayed release for BMP-2 [19,20,21,36,37,38,39]. As a result, the varied loads of PDGF-BB and BMP-2 in the presently employed ALG MPs and core-shell MPs may not be high enough to significantly increase the concentration gradient of PDGF-BB or BMP-2 in their corresponding MPs, leading to insignificant changes in release rates of their matched composite gels.

**Figure 5:**
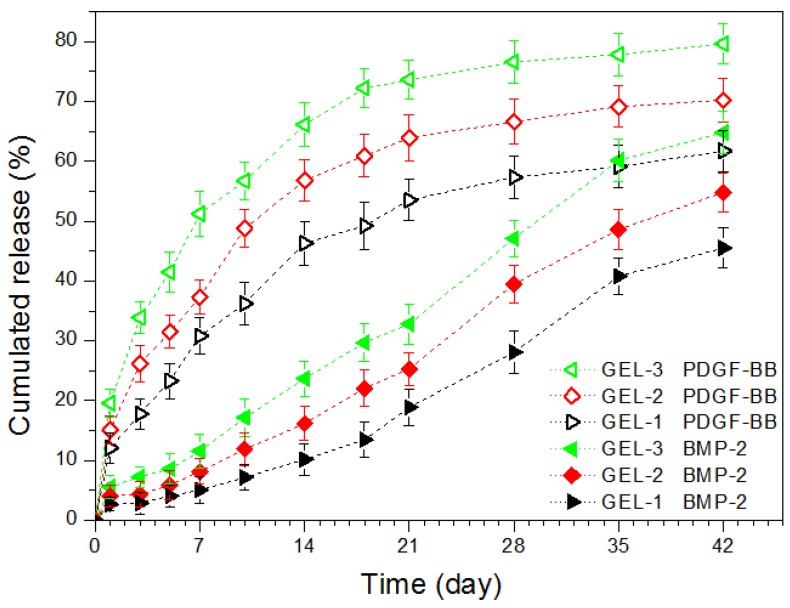
Cumulative release profiles for composite gels containing varied amounts of growth factors and microspheres.

Taking into account the lack of effective regulation related to above produced gels, an attempt was further made to formulate another set of composite gels in which the amount of the embedded MPs and the factor load were both varied in order to effectively modulate the release rate of the loaded factors, and the relevant results are represented in Figure 5 and Table 5.

The curves in Figure 5 indicate that these composite gels showed capacities in administering the release of both PDGF-BB and BMP-2 in a temporally separated manner while effectively regulating the release rates of the factors. In the present situations, the factor load for the two types of MPs was maintained as a constant, but the embedded amount of MPs in the gels was changed. When a higher amount of MPs was dispersed in the gel matrix, the contact area between the MPs and the gel matrix will increase, which would promote more loaded PDGF-BB and BMP-2 to diffuse into the gel matrix and result in their faster release from the gels when compared with those gels containing a lower amount of MPs. It is worth pointing out that the release patterns shown in Figure 5 rely on the initial factor load in MPs. The initial factor load for the two types of MPs was devised as around 85 (ng/mg) for PDGF-BB and 152 (ng/mg) for BMP-2 (see Table 5) in order to make comparisons between the gel set illustrated in Table 4 and the gel set presented in Table 5. In fact, by changing the initial factor load within an appropriate range for two kinds of MPs or increasing the total amount of the embedded MPs up to an upper limit of about 2.0% (*w*/*v*), the release rate of PDGF-BB and BMP-2 can be further regulated in a wider range and in different ways. Results in Figure 5 and Table 5 suggest that these composite gels have potential for certain bone repair and regeneration where the sequential administration of PDGF-BB and BMP-2 is required while the applied dosages of the factors need to be well controlled. Based on the details described in the experimental section for the preparation of different types of MPs and the resulting composite gels, it can be inferred that the presently developed gel systems can actually function as a platform for sequentially delivering different signal molecules or hydrophilic drugs provided that the adopted molecules or drugs could be efficiently loaded into the employed MPs.

### 2.4. Bioactivity Assessment of Released Factors

**Figure 6:**
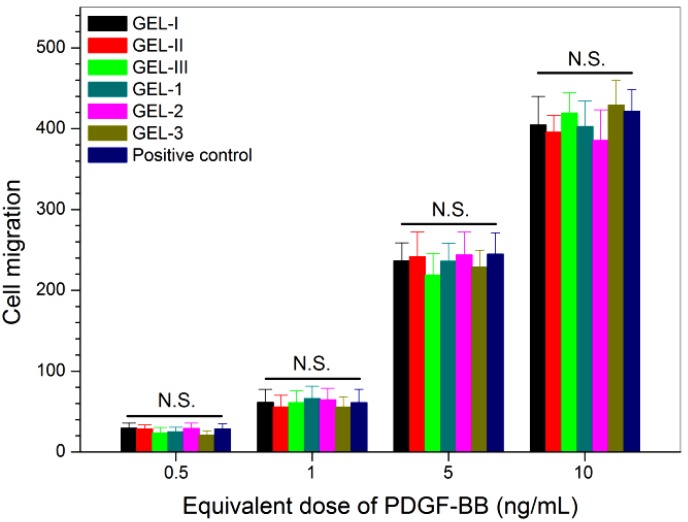
Chemotactic response of Balb/c 3T3 cells to the PDGF-BB released from different composite gels (culture time: 4 h; see Table 4 and Table 5 for the composition of gels; control: free PDGF-BB; N.S.: no significance).

One of important biological functions of PDGF-BB is its chemotactic activity [40,41]. Hence, the released PDGF-BB was compared with free PDGF-BB to see their ability for inducing the migration of Balb/c 3T3 cells using Boyden-type chambers [42]. Since the release medium contained both PDGF-BB and BMP-2, the positive control employed for these measurements was prepared by mixing both free PDGF-BB and free BMP-2, and the applied amount of free PDGF-BB and free BMP-2 was precisely matched with the amount of equivalent PDGF-BB and BMP-2 in the release medium. By doing so, the effect of the released BMP-2 on the migration of Balb/c 3T3 cells can be deduced. The measured numbers of migrated cells are graphed in Figure 6. The results show that the chemotactic response of Balb/c 3T3 cells to the released PDGF-BB was dependent on the applied equivalent PDGF-BB doses, and PDGF-BB-induced cell migration was detected even though the dose of the applied PDGF-BB was as low as 0.5 ng/mL. The number of migrated cells increased as the dose of applied PDGF-BB rose; and there were no significant differences (*p* > 0.05) in the number of the migrated cells between the released PDGF-BB group and the free PDGF-BB group. The results shown in Figure 6 signify that the bioactivity of the loaded PDGF-BB in all these composite gels is well preserved.

**Figure 7:**
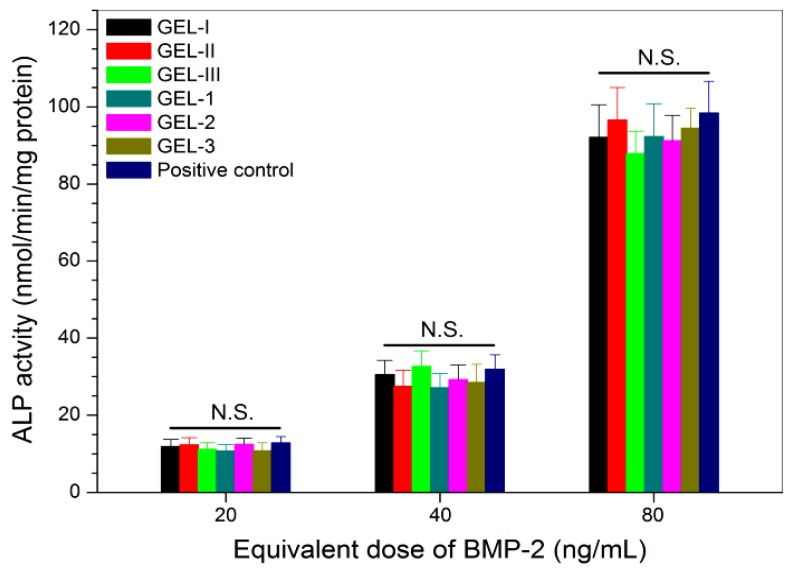
Alkaline phosphatase activity of C2C12 cells that were cultured with BMP-2 released from different composite gels (culture time: 7 days; see Table 4 and Table 5 for the composition of gels; control: free BMP-2; N.S.: no significance).

BMP-2 is known to be capable of reprograming some types of myoblasts through the transdifferentiation pathway [43,44]. In this study, a biological assay based on the BMP-2-induced ALP synthesis in C2C12 cells was used for detecting the activity of the released BMP-2, since BMP-2 has the ability to reprogram C2C12 cells to an osteogenic lineage [37,43,44,45]. Similar to the case of PDGF-BB activity testing, the positive control used here was also prepared by mixing both free BMP-2 and free PDGF-BB while controlling the applied amount of free PDGF-BB and free BMP-2 as the same as that of the equivalent PDGF-BB and BMP-2 in the release medium. The obtained data are depicted in Figure 7. The bar-graphs show a dose-dependency trend for the BMP-2-induced ALP activity in C2C12 cells, and the released BMP-2 from all composite gels had almost equal efficiency in inducing ALP activity when compared with the free BMP-2 without significant difference (*p* > 0.05); the activity of the loaded BMP-2 is effectively retained.

In the present study, CaCl_2_ and TPP, two types of ionic crosslinkers, were used to prepare the PDGF-BB-loaded ALG MPs and the BMP-2-loaded core-shell MPs, respectively. The preparation of these MPs was conducted using an emulsion technique under mild processing conditions, and hence, the activity of the loaded PDGF-BB or BMP-2 in MPs can be effectively preserved. On the other hand, the gel matrix contains highly biocompatible CH and GP components, and its preparation does not involved adverse factors such as covalent crosslinkers, harsh organic solvents, and unsuitable processing temperature and pH,; thus, the CH/GP matrix would not impair the bioactivity of PDGF-BB or BMP-2 molecules that diffuse from the MPs and pass through the gel matrix to reach the release medium. Based on the above-presented results, it can be concluded that the presently developed composite gels are reliable for sustaining the release of PDGF-BB or BMP-2 in a sequential manner with tunable release rates while effectively preserving the bioactivity of the loaded PDGF-BB and BMP-2.

## 3. Materials and Methods

### 3.1. Materials

CH powder (deacetylation degree: >90%; viscosity-average molecular weight: ca. 6.7 × 10^4^) and sodium alginate (ALG, low viscosity, 180–220 mpa.s of 2% solution) were supplied by Aladdin Inc. (Shanghai, China). BMP-2 and BMP-2 ELISA Kits were purchased from PeproTech Inc. (Rocky Hill, CT, USA) and Abcan Inc. (Shanghai, China), respectively. PDGF-BB and PDGF-BB ELISA Kits were bought from R&D Systems Inc. (Minneapolis, MN, USA) and IBL-America Inc. (Minneapolis, MN, USA). All other reagents and chemicals were of analytical grade and purchased from Sinopharm (Shanghai, China).

Chitosan-polylactide (CH-PLA) copolymers were synthesized using phthaloylchitosan (PHCS) as the intermediate, following reported methods [30,31]. The CH-PLA with a PLA content of around 31 wt% was selected for the preparation of CH-PLA MPs.

Fluorescein isothiocyanate (FITC) conjugated ALG (FITC-ALG) with a FITC content of about 1.86 wt% was synthesized using a reported method [46], and it was used together with unlabeled ALG for imaging the coating layer of CH-PLA MPs.

### 3.2. Preparation of PDGF-BB Loaded ALG Microspheres

PDGF-BB loaded ALG MPs were produced using an emulsification method similar to that described elsewhere [47,48]. In brief, to a 1.5% (*w*/*v*) ALG solution in deionized water, a given amount of PDGF-BB solution was introduced with gentle stirring for 5 min. The mixture was then added to an iso-octanol system containing 5% (*v*/*v*) Span-80 by using a 16 G needle while being dispersed at 2000 rpm for 20 min using a homogenizer (PRO200, PRO Scientific, Buffalo Grove, IL, USA). The volume ratio of organic phase to aqueous phase for this mixture was 8. After that, a CaCl_2_ aqueous solution (1.0 M) with a feed weight ratio of CaCl_2_ to ALG at 6 was slowly added to the mixture with stirring for an additional 30 min. The mixture was then processed with 2-propanol for 5 min to harden the resulting MPs. The prepared PDGF-BB-loaded ALG MPs were retrieved by centrifugation, further washed with ethanol and deionized water, and lyophilized. Blank ALG MPs with similar mean size and surface charging features were also prepared using the same protocol and named BM-I (see Table 1).

### 3.3. Preparation of BMP-2 Loaded Core-Shell Microspheres

BMP-2-loaded core-shell MPs were produced via a two-step method. BMP-2 was first encapsulated into CH-PLA MPs with an emulsification method using sodium tripolyphosphate (TPP) as a crosslinker, and the resulting BMP-2-encapsulated CH-PLA MPs were then coated with ALG to achieve BMP-2 loaded core-shell MPs with a negatively charged surface nature. In the first step, the selected CH-PLA was dissolved in 1% acetic acid aqueous solution to prepare a CH-PLA solution (1.0%, *w*/*v*). To this solution, a given amount of BMP-2 was introduced, and the mixture was emulsified with n-octanol containing a 4% (*w*/*v*) surfactant of hydrogenated caster-oil-60 using a homogenizer at 3000 rpm for 20 min. The volume ratio of organic phase to aqueous phase was 8. Thereafter, a TPP aqueous solution (10%, *w*/*v*) was slowly added into the emulsion (feed weight ratio of TPP to CH-PLA: 5) and homogenized at 3000 rpm for an additional 30 min. The BMP-2-encapsulated CH-PLA MPs were collected by centrifugation and further washed with 2-propanol, ethanol, and water. These MPs were either kept in a wet state or lyophilized for further use. Blank CH-PLA MPs with a relatively small mean size in comparison to that of BM-I MPs were also prepared with the same method and named BM-II (see Table 1).

In the second step, wet BMP-2-encapsulated CH-PLA MPs (200 mg) were dispersed in 4 mL of the coating solution (1.0%, *w*/*v*) that was prepared by mixing unlabeled ALG and FITC-ALG at a weight ratio of 5:1 in deionized water. The resulting mixture was gently stirred for 1.5 h. The BMP-2 loaded core-shell MPs were collected by centrifugation, and the ALG layer of MPs was further solidified by using a 5% CaCl_2_ aqueous solution for 5 min. Finally, the MPs were washed with deionized water and lyophilized. Blank core-shell MPs with a mean size and surface charging nature respectively similar to that for BM-I MPs were also prepared following the same protocol and named BM-III (see Table 1).

### 3.4. Characterization

The content of PLA in CH-PLAs was determined with an elemental analyzer (Vario EL III, Elementar, Hanau, Germany) by measuring the content of C, H, and N in CH-PLAs. The sizes and shapes of MPs were viewed using a scanning electron microscope (SEM, Quanta 200, FEI, Eindhoven, The Netherlands). The mean size of MPs was calculated by averaging the measured diameters of 200 different MPs in each SEM image. The core-shell MPs having a FITC-labeled shell were viewed using a confocal microscope (Leica TCS SP5, Leica Microsystems, Buffalo Grove, IL, USA; λ_ex_ = 488 nm; λ_em_ = 519 nm). Zeta (ζ) potential of MPs was determined by electrophoretic light scattering measurements (Zetasizer Nano ZS, Malvern, UK).

Different types of factor-loaded MPs were cryogenically ground into powder using a freezer mill (SPEX 6750, Metuchen, NJ, USA). For each sample, a precisely weighed amount of powder was extracted in PBS at 37 °C for 2 h using a shaking table. The extraction of the same powder was repeated twice, and the supernatants were collected by centrifugation. The initial load of PDGF-BB or BMP-2 in the matched MPs was determined using their respective ELISA Kits. The encapsulation efficiency (EE) of MPs and factor load (FL) was calculated by the following equations:

EE(%) = [M_0_/M_1_] × 100% (1)

FL = M_0_/M (2)

where M_0_ is the measured amount of the loaded factor in MPs, M_1_ refers to the feed-in amount of factor, and M denotes the mass of MPs.

Weighed dry MPs (W_d_) were immersed in a PBS solution at 37 °C for 3 h. After that, they were transferred into thimbles for removal of excess water by centrifugation at 2000 rpm for 1 min. The weight (W_s_) of swollen MPs was measured, and their swelling index (SI) was calculated as follows:

SI(%) = [(W_s_ − W_d_)/W_d_] × 100% (3)

The main parameters for blank MPs and factor-loaded MPs are presented in Table 1 and Table 2, respectively.

### 3.5. Preparation of Composite Solutions

Composite solutions without loading any factor were prepared using CH, GP, BM-I, and BM-III MPs (see Table 1) in various proportions, and they were used for rheological measurements of resulting composite gels. Relevant data for formulations of these gels are summarized in Table 3. Similarly, composite solutions containing varied amounts of factors were also prepared by embedding two types of factor-encapsulated MPs into the CH/GP gel, and formulations for these factor-incorporated composite gels are illustrated in Table 4 and Table 5.

Gelation time was estimated with a tube-titled method. In a typical measurement process, 2.0 mL of the prepared composite solution was first stirred in an ice/water bath for 5 min and then added to a glass vial. The vial was incubated at 37 °C in a water bath for examining flowability of the solution by titling or inverting the vial every 20 s. Gelation time was recorded starting from the time point for the vial incubation and ending at the moment when the solution stopped flowing.

### 3.6. Rheological Measurement

Rheological measurements of composite solutions were conducted using a rheometer (Kinexus Pro KNX2100, UK) equipped with a parallel-plate sample holder. The storage modulus (G′) and loss modulus (G″) of samples were measured at a constant strain amplitude of 1% and a frequency of 1.0 Hz. Temperature–dependence spectra of G′ and G″ were recorded in a range between 25 and 45 °C at an elevating rate of 1 °C/min, and the incipient gelling temperatures (T_i_) of composite solutions was determined from the intersection point of G′ and G″.

### 3.7. In Vitro Release of Factors

One of the measurements is described briefly as follows. A CH/GP solution (0.5 mL) containing a prescribed amount of MPs (see Table 4 and Table 5) was filled into a mold (cylinder with a lid at one end; diameter: 8 mm), and the mold was incubated at 37 °C for 20 min for the gel formation. The resulting gel sample was placed in a vial, followed by the addition of 4 mL of PBS. The vials were shaken on a swirling table at 37 °C and 60 rpm. At predetermined time intervals, 0.5 mL of medium was withdrawn and the same volume of fresh buffer was replenished. The released PDGF-BB and BMP-2 were detected using the corresponding ELISA Kits.

### 3.8. Bioactivity Assessment

Balb/c 3T3 cells and C2C12 cells (both types of cells were obtained from Sixin Biological Technology Inc., Shanghai, China) were expanded in DMEM supplemented with 10% fetal bovine serum, 100 U/mL penicillin, and 100 μg/mL streptomycin at 37 °C in a 5% CO_2_ atmosphere. The culture medium was refreshed twice weekly until cell confluence. The harvested cells were resuspended in media for further use.

The activity of the released PDGF-BB was detected by assessing whether it has chemotactic effects on Balb/c 3T3 cells [41,42]. The chemotaxis assay was performed using a 48-well chamber that consists of upper and lower compartments separated by porous membrane filters (pore size: 8 μm). The filters were coated with a type-I collagen solution (100 μg/mL) for one day before cell seeding. After that, Balb/c 3T3 cells were seeded into wells (1.5 × 10^4^ cells/well) in the upper compartment, and the wells in the lower compartment were filled with diluents of released medium. After a 4-h incubation, the chamber was disassembled, and non-migrated cells on the upper side of filters were scraped using a rubber wiper. The migrated cells on the lower side of filters were fixed in formalin and stained with toluidine blue for counting. Since the released medium contained both PDGF-BB and BMP-2, free PDGF-BB and free BMP-2, both of which are identical to the equivalent amount of factors in the applied release medium, were used together as a positive control.

The activity of the released BMP-2 was detected by evaluating its ability to induce alkaline phosphatase (ALP) activity in C2C12 cells [43,44]. C2C12 cells were seeded in 24-well culture plates (5 × 10^4^ cells/well) with DMEM containing 2% fetal bovine serum and 1% penicillin/streptomycin and incubated at 37 °C in a 5% CO_2_ atmosphere for 6 h. Thereafter, cells were incubated with the released medium for 7 days with the medium changed every two days. Similarly, the positive control was composed of free BMP-2 and free PDGF-BB that were respectively identical to the equivalent amount of factors in the release medium used for the treatment of C2C12 cells. These C2C12 cells were lysed with 0.5% Triton X-100 in PBS and exposed to freeze-thaw treatments. The lysates were centrifuged at 12,000 rpm at 4 °C to collect supernatants. ALP activity in supernatants was detected photometrically at 405 nm using an ALP detection Kit (Beyotime, Shanghai, China).

### 3.9. Statistical Analysis

Data were shown as mean ± standard deviation. The statistical difference between groups was determined by using one-way ANOVA. Differences were considered to be statistically significant at a level of *p* < 0.05.

## 4. Conclusions

Chitosan (CH)-polylactide (PLA) copolymers with free amino groups and soluble characters in water-based solvents were found to be suitable for fabricating ion-crosslinked core-shell microspheres (MPs) that can carry BMP-2. PDGF-BB-loaded alginate MPs were also successfully prepared by using calcium ions as a crosslinker. Composite gels that were constructed by embedding the PDGF-BB-loaded alginate MPs and the BMP-2-loaded core-shell MPs into the CH/glycerophosphate (GP) hydrogel at optimal compositional proportions were found to be injectable at ambient temperatures and showed phase transition features near physiological temperature and pH. The optimal MP-embedded CH/GP gel systems were demonstrated to have abilities to administer the release of PDGF-BB early in the combination utility of PDGF-BB and BMP-2, and the release patterns of these factors could be regulated by the embedded amount of different factor-loaded MPs and the initial factor load in these MPs. In addition, these composite gels were capable of effectively maintaining the bioactivity of the loaded factors. The optimized composite gels have potential for applications in bone repair by sequentially providing growth-promoting cues for bone regeneration.

## Figures and Tables

**Table 1:** Parameters of blank microspheres ^a^.

Sample Name	Mean Size (μm)	ζ (mV)	SI (%)
BM-I ^a^	13.6 ± 1.59	−19.1 ± 1.82	86.4 ± 9.17
BM-II ^b^	7.8 ± 1.16	29.5 ± 2.46	38.1 ± 3.26
BM-III ^c^	14.1 ± 1.72	−17.8 ± 1.93	41.5 ± 4.43

^a^ Blank ALG MPs; ^b^ Blank CH-PLA MPs; ^c^ Blank ALG-coated CH-PLA MPs.

**Table 2:** Parameters of factor-encapsulated microspheres.

Sample Name	PDGF-BB	BMP-2	Mean Size (μm)	ζ (mV)	EE (%) ^c^	FL (μg/mg) ^d^	SI (%)
LM-1 ^a^	+	−	14.7 ± 1.75	−19.6 ± 1.79	58.6 ± 3.72	4.2 ± 0.31	83.2 ± 9.53
LM-2 ^b^	−	+	15.3 ± 1.69	−18.1 ± 1.64	87.3 ± 4.15	8.6 ± 0.48	42.3 ± 4.28

^a^ LM-1 MPs were prepared by loading PDGF-BB into ALG MPs and using exactly the same protocol applied to BM-I MPs (see Table 1); ^b^ LM-2 MPs were prepared by first incorporating BMP-2 into CH-PLA MPs, and then, coating the BMP-2-incorporated CH-PLA MPs with an ALG layer. For the convenience of description, LM-2 MPs are often referred to as BMP-2 loaded core-shell MPs in the text or in table; ^c^ The encapsulation efficiency (EE) was optimized by altering the fed amount of the factor while keeping the composition of MPs and processing conditions constant; ^d^ Factor load (FL) corresponded to the optimal EE.

**Table 3:** Parameters for hydrogels without loading growth factor ^a^.

Sample Name	CH (*w*/*v*%)	Blank ALG MPs (*w*/*v*%)	Blank Core-Shell MPs (*w*/*v*%)	pH	Gelation Time at 37 °C (s) ^f^	T_i_ (°C) ^g^
GL-0 ^b^	2.0	−	−	7.02 ± 0.06	620 ± 16	35.8 ± 1.16
GL-1 ^c^	2.0	1.2	−	7.09 ± 0.08	530 ± 11	35.1 ± 1.29
GL-2 ^d^	2.0	−	1.2	7.13 ± 0.09	515 ± 10	34.6 ± 1.05
GL-3 ^e^	2.0	0.6	0.6	7.15 ± 0.07	525 ± 10	34.2 ± 1.13

^a^ Glycerophosphate (GP) content was 5.0 (*w*/*v*%). ^b^ This was chitosan (CH)/GP gel and used as control. ^c^ This gel was prepared by using BM-I MPs. ^d^ This gel was prepared by using BM-III MPs. ^e^ This gel was prepared by using both BM-I and BM-III MPs. ^f^ Gelation time was determined by using a tilting or inverting method. ^g^ T_i_ indicates the incipient gelation temperature, and it was determined via the temperature–dependence functions of G′ and G″.

**Table 4:** Parameters for hydrogels containing different amounts of growth factors but the same amount of microspheres ^a^.

Sample Name	CH (*w*/*v*%)	PDGF-BB Loaded ALG MPs		PBGF-BB (ng/mL)	BMP-2 Loaded Core-Shell MPs		BMP-2 (ng/mL)
		PDGF-BB Content in MPs (ng/mg) ^b^	MPs (*w*/*v*%)		BMP-2 Content in MPs (ng/mg) ^c^	MPs (*w*/*v*%)	
GEL-I	2.0	46.2 ± 3.17	0.4	172.6 ± 14.37	78.3 ± 6.04	0.4	305.2 ± 23.15
GEL-II	2.0	85.7 ± 5.69	0.4	331.2 ± 21.62	152.1 ± 12.76	0.4	612.8 ± 37.91
GEL-III	2.0	124.8 ± 9.21	0.4	504.7 ± 29.31	231.6 ± 19.13	0.4	910.6 ± 41.63

^a^ GP content was 5.0 (*w*/*v*%). ^b^ These MPs were prepared following the protocol similar to that applied to LM-1 MPs but loaded with different amounts of PDGF-BB (see Table 2). ^c^ These MPs were prepared following the protocol similar to that applied to LM-2 MPs but loaded with different amounts of BMP-2 (see Table 2).

**Table 5:** Parameters for hydrogels containing varied amounts of growth factors and microspheres ^a^.

Sample Name	CH (*w*/*v*%)	PDGF-BB Loaded ALG MPs		PBGF-BB (ng/mL)	BMP-2 Loaded Core-Shell MPs		BMP-2 (ng/mL)
		PDGF-BB Content in MPs (nm/mg) ^b^	MPs (*w*/*v*%)		BMP-2 Content in MPs (ng/mg) ^c^	MPs (*w*/*v*%)	
GEL-1	2.0	85.7 ± 5.69	0.2	165.3 ± 13.29	152.1 ± 12.76	0.2	310.6 ± 22.73
GEL-2	2.0	85.7 ± 5.69	0.4	331.2 ± 21.62	152.1 ± 12.76	0.4	612.8 ± 37.91
GEL-3	2.0	85.7 ± 5.69	0.6	512.6 ± 30.46	152.1 ± 12.76	0.6	916.2 ± 43.18

^a^ GP content was 5.0 (*w*/*v*%). ^b^ These MPs were prepared following the protocol similar to that applied to LM-1 MPs (see Table 2). ^c^ These MPs were prepared following the protocol similar to that applied to LM-2 MPs (see Table 2).
